# Increased risk of cerebrovascular mortality in head and neck cancer survivors aged ≥ 65 years treated with definitive radiotherapy: a population-based cohort study

**DOI:** 10.1186/s13014-021-01913-3

**Published:** 2021-09-20

**Authors:** Qing-Song He, Zhen-Ping Wang, Zhao-Jun Li, Ping Zhou, Chen-Lu Lian, San-Gang Wu, Si-Fang Chen

**Affiliations:** 1 Department of Neurology, The First Affiliated Hospital of Xiamen University, Xiamen, 361003 People’s Republic of China; 2 Department of Radiology, Hainan General Hospital (Hainan Affiliated Hospital of Hainan Medical University), Haikou, 570311 People’s Republic of China; 3 Department of Radiation Oncology, Hainan General Hospital (Hainan Affiliated Hospital of Hainan Medical University), Haikou, 570311 People’s Republic of China; 4 Department of Radiation Oncology, The First Affiliated Hospital of Xiamen University, Xiamen, 361003 People’s Republic of China; 5 Department of Neurosurgery, the First Affiliated Hospital of Xiamen University, Xiamen, 361003 People’s Republic of China

**Keywords:** Head and neck cancer, Radiotherapy, Cerebrovascular mortality, Older, Survivors

## Abstract

**Background:**

To investigate the relationship between radiotherapy (RT) and the risk of cerebrovascular mortality (CVM) in head and neck cancer (HNC) survivors aged ≥ 65 years.

**Methods:**

Patients with HNC survivors aged ≥ 65 years diagnosed between 2000 and 2012 were included from the Surveillance, Epidemiology, and End Results database. Kaplan–Meier analysis, Log-rank tests, and Cox proportional-hazards regression models were performed for statistical analyses.

**Results:**

We included 16,923 patients in this study. Of these patients, 7110 (42.0%) patients received surgery alone, 5041 (29.8%) patients underwent RT alone, and 4772 (28.2%) patients were treated with surgery and RT. With a median follow-up time of 87 months, 1005 patients died with cerebrovascular disease. The 10-years CVM were 13.3%, 10.8%, and 11.2% in those treated with RT alone, surgery alone, and surgery plus RT, respectively (P < 0.001). The mean time for CVM was shorter in RT alone compared to surgery alone and surgery plus RT (52 months vs. 56–60 months). After adjusting for covariates, patients receiving RT alone had a significantly higher risk of developing CVM compared to those receiving surgery alone (hazard ratio [HR] 1.703, 95% confidence interval [CI] 1.398–2.075, P < 0.001), while a comparable risk of CVM was found between those treated with surgery alone and surgery plus RT (HR 1.106, 95% CI 0.923–1.325, P = 0.274). Similar trends were found after stratification age at diagnosis, gender, tumor location, and marital status.

**Conclusions:**

Definitive RT but not postoperative RT can increase the risk of CVM among older HNC survivors. Long-term follow-up and regular screening for CVD are required for HNC patients who received definitive RT to decrease the risk of CVM.

## Background

Head and neck cancer (HNC) accounts for approximately 3–5% of newly diagnosed cancers each year [1]. Surgery and radiotherapy (RT) remain the cornerstone for local treatment of HNC, and postoperative RT should be administrated in those with high-risk recurrence factors [2, 3]. However, several studies have shown that receiving RT increases the risk of cerebrovascular disease (CVD) or cerebrovascular mortality (CVM) in HNC patients [4–6], and the excess risk of CVD among HNC survivors increases with attained age [7]. It might be due to the chronic damage of the vasculatures by the use of RT in the head and neck region [8, 9]. Therefore, once RT has been completed, physicians, patients, and even insurance practitioners should jointly evaluate the risk of CVD or CVM.

Although several prior studies have assessed the CVD or CVM risk after RT, several limitations should be noticed in these studies [4, 6, 10, 11], including no distinction between definitive and adjuvant RT [6, 10], short follow-up time for survivors [4], and excluded laryngeal cancer patients [4]. Thus, a well-designed study with long-term follow-up is needed to reflect the current relationship between RT and CVD or CVM. In light of this, we used a large cohort of HNC survivors from the Surveillance, Epidemiology, and End Results (SEER) program to analyze the relationship between RT and the risk of CVM.

## Material and methods

### SEER database and patients

The SEER database is the authoritative source of information regarding the incidence and survival of cancer in the United States (US), which covers approximately 35 percent of the US population [12]. The study cohort consisted of patients with HNC survivors aged ≥ 65 years who had undergone surgery, RT, or surgery plus adjuvant RT between 2000 and 2012. Patients were excluded if they (1) had no positive histology, (2) had died with other causes (non-cerebrovascular mortality), (3) had no anti-cancer local treatment or unknown the local treatment status, (4) had non-beam RT technologies including radioisotopes and radioactive implants, (5) had follow-up time ≤ 12 months. Since this SEER program includes the de-identified data of patients, the current study was exempted from approval by the Institutional Review Board.

### Measurements

We included the following demographic, clinicopathological variables, and survival outcomes of each patient: age, gender, race/ethnicity, tumor grade, histology, SEER stage, tumor location, marital status. Treatment variables, including surgery, definitive RT, adjuvant RT, and chemotherapy were also included. The primary outcome in the study was CVM, which was defined as the time from the initial diagnosis of HNC to death from CVD.

### Statistical analysis

We compared the risk of CVM among the three treatment groups including surgery alone, RT alone, and surgery plus RT. The differences in categorical variables among the three treatment cohorts were tested by Pearson's chi-squared test. The rate of CVM was estimated by the Kaplan–Meier method and compared by the log-rank test. Multivariate Cox proportional hazards models were used to determine the risk factors associated with CVM. Sensitivity analyses focused on age, gender, tumor sites, and marital status were performed. Statistical analyses were conducted with SPSS statistical software (version 22.0, IBM Corporation, Armonk, NY, USA). A P value of < 0.05 was considered statistically significant.

## Results

### Patient characteristics

**Fig. 1:**
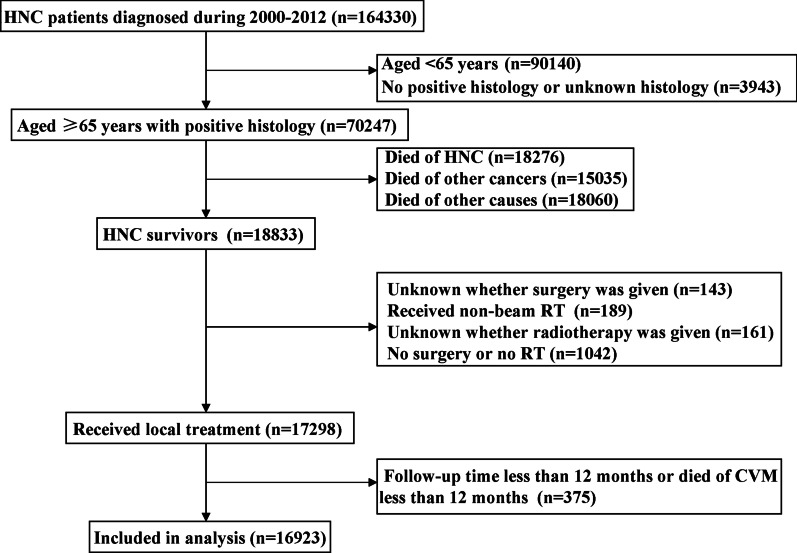
The patient selection flowchart of the study

We included 16,923 patients in this study (Table 1). Figure 1 depicts the patient selection flowchart of the study. The Median age at the time of diagnosis was 71 years (range, 65–100 years), 67.2% (n = 11,380) were male, 79.9% (n = 13,518) were Non-Hispanic White and 82.9% (n = 14,031) had squamous histology. At diagnosis, 58.3% (n = 9869), 32.4% (n = 5479), 6.6% (n = 1111) of patients had localized, regional, and distant stage, respectively. Most cancers developed in the oral cavity (35.3%) and larynx (26.4%). Of these patients, 7110 (42.0%) patients received surgery alone, 5041 (29.8%) patients underwent RT alone, and 4772 (28.2%) patients were treated with surgery and RT. Most patients with tumors located in the oral cavity had undergone surgery alone (57.1%), whereas patients with tumors developed in the oropharynx (48.6%), nasopharynx (77.6%), hypopharynx (68.6%), and larynx (50.8%) were more likely to treat with RT alone. In addition, patients with tumors developed in the salivary gland (55.8%), and nasal cavity and sinus (45.5%) were more likely to received surgery and RT. Moreover, only 23.5% (n = 3974) of patients received chemotherapy.

**Table 1:** Patient baseline characteristics by local treatment groups

Variables	n	Surgery alone (%)	RT alone (%)	Surgery + RT (%)	P
Age (years)					
65–69	6852	2424 (34.1)	2371 (47.0)	2057 (43.1)	< 0.001
70–74	4652	1912 (26.9)	1430 (28.4)	1310 (27.5)	
≥ 75	5419	2774 (39.0)	1240 (24.6)	1405 (29.4)	
Gender					
Male	11,380	4129 (58.1)	4009 (79.5)	3242 (67.9)	< 0.001
Female	5543	2981 (41.9)	1032 (20.5)	1530 (32.1)	
Race/ethnicity					
Non-Hispanic White	13,518	5866 (82.5)	3903 (77.4)	3749 (78.6)	< 0.001
Non-Hispanic Black	1056	294 (4.1)	434 (8.6)	328 (6.9)	
Hispanic (all races)	1197	473 (6.7)	366 (7.3)	358 (7.5)	
Other	1049	414 (5.8)	310 (6.1)	325 (6.8)	
Unknown	103	63 (0.9)	28 (0.6)	12 (0.3)	
Grade					
Well differentiated	3256	2095 (29.5)	508 (10.1)	653 (13.7)	< 0.001
Moderately differentiated	6139	2571 (36.2)	1827 (36.2)	1741 (36.5)	
Poorly/undifferentiated	3605	742 (10.4)	1415 (28.1)	1448 (30.3)	
Unknown	3923	1702 (23.9)	1291 (25.6)	930 (19.5)	
Histology					
Squamous histology	14,031	5584 (78.5)	4806 (95.3)	3641 (76.3)	< 0.001
Non-squamous histology	2892	1526 (21.5)	235 (4.7)	1131 (23.7)	
SEER stage					
Localized	9869	5586 (78.6)	2124 (42.1)	2159 (45.2)	< 0.001
Regional	5479	1110 (15.6)	2282 (45.3)	2087 (43.7)	
Distant	1111	176 (2.5)	488 (9.7)	447 (9.4)	
Unknown	464	238 (3.3)	147 (2.9)	79 (1.7)	
Tumor location					
Oral cavity	5966	3405 (47.9)	1291 (25.6)	1270 (26.6)	< 0.001
Oropharynx	1566	195 (2.7)	761 (15.1)	610 (12.8)	
Nasopharynx	361	19 (0.3)	80 (5.6)	62 (1.3)	
Hypopharynx	338	37 (0.5)	232 (4.6)	69 (1.4)	
Nasal cavity and sinus	747	309 (4.3)	98 (1.9)	340 (7.1)	
Salivary gland	1772	761 (10.7)	22 (0.4)	989 (20.7)	
Larynx	4471	856 (21.5)	2273 (45.1)	1342 (28.1)	
Other	1702	1528 (12.0)	84 (1.7)	90 (1.9)	
Chemotherapy					
No	12,949	7040 (99.0)	2369 (47.0)	3540 (74.2)	< 0.001
Yes	3974	70 (1.0)	2672 (53.0)	1232 (25.8)	
Marital status					
Married	10,397	4108 (57.8)	3211 (63.7)	3078 (64.5)	< 0.001
Divorce	1395	484 (6.8)	514 (10.2)	397 (8.3)	
Single	1559	600 (8.4)	506 (10.0)	453 (9.5)	
Widowed	2308	1114 (15.7)	548 (10.9)	646 (13.5)	
Unknown	1264	804 (11.3)	262 (5.2)	198 (4.1)

### The incidence of CVM

With a median follow-up time of 87 months (range, 13–203 months), 1005 patients died with CVD: 423 (42.1%) in the surgery alone cohort, 324 (32.2%) in the RT alone cohort, and 258 (25.7%) in the surgery plus RT cohort. The 3-, 5-, 10-, and 15-years of CVM was 1.8%, 3.3%, 7.2%, and 11.5%, respectively.

**Fig. 2:**
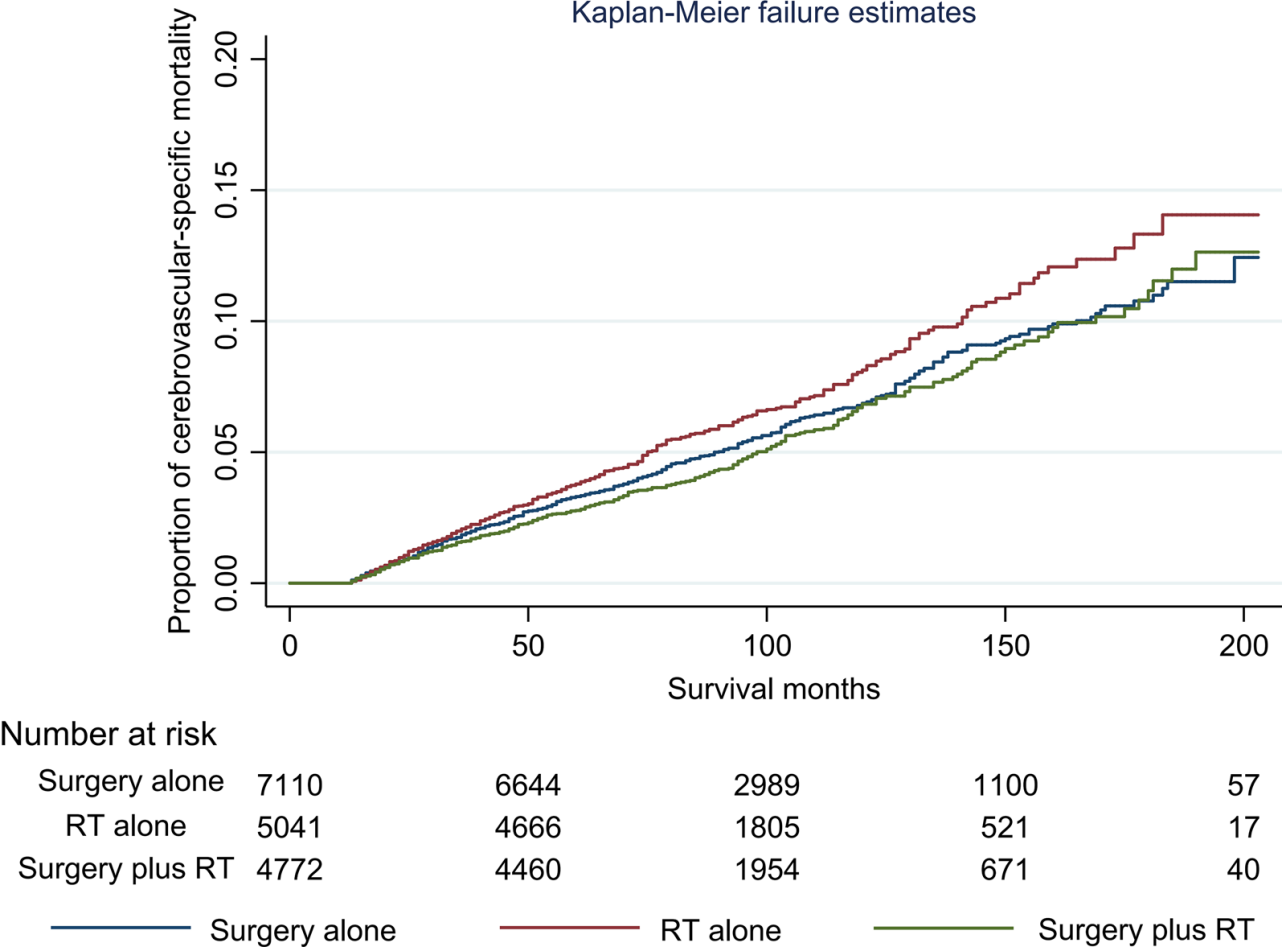
Cumulative incidence of cerebrovascular-specific mortality by local treatment groups

The risk of CVM was highest in patients receiving RT alone than those receiving surgery alone or surgery plus RT (P < 0.001). The 5-years CVM was 8.1% in patients with RT alone compared with 6.9% in the surgery alone cohort and 6.8% in the surgery plus RT cohort. The corresponding 10-years CVM were 13.3%, 10.8%, and 11.2%, respectively (Fig. 2; Table 2). The mean time for CVM was shorter in RT alone compared to surgery alone and surgery plus RT (52 months vs. 56–60 months).

**Table 2:** Actuarial incidence of cerebrovascular mortality by local treatment groups

Treatment	3-year (%)	5-years (%)	10-years (%)	15-years (%)	P ^a^	P ^b^	P ^c^
Surgery alone	1.7	3.3	6.9	10.8	0.024	0.323	0.002
RT alone	2.1	3.8	8.1	13.3			
Surgery plus RT	1.6	2.8	6.8	11.2			

a indicates surgery alone versus RT alone; b indicates surgery alone versus surgery plus RT; c indicates RT alone versus surgery plus RT

### Risk factors associated with CVM

We used the Cox proportional hazard regression model to investigate the risk factors associated with CVM (Table 3). After adjustment by age, gender, race/ethnicity, histology, SEER stage, grade, tumor location, chemotherapy, and marital status, our results showed that patients receiving RT alone had a significantly higher risk of developing CVM compared to those receiving surgery alone (hazard ratio [HR] 1.703, 95% confidence interval [CI] 1.398–2.075, P < 0.001), which comparable risk of CVM was found between those treated with surgery alone and surgery plus RT (HR 1.106, 95% CI 0.923–1.325, P = 0.274). In addition, age at diagnosis, chemotherapy, race/ethnicity, tumor grade, SEER stage, and marital status were also the independent risk factors associated with cerebrovascular mortality. Similar trends were found after stratification by age at diagnosis, gender, tumor location, SEER stage, and marital status (Table 3). In patients without chemotherapy, we also found a significant effect of definitive RT on the increase of CVM. As only 70 patients receiving chemotherapy in the surgery alone group, we used surgery plus RT as a reference in the final Cox proportional hazard regression model. We also found a significant effect of definitive RT on the increase of CVM compared to those receiving surgery plus RT (HR 1.779, 95% CI 1.210–2.615, P = 0.003), and comparable risk of CVM between those treated with surgery alone and surgery plus RT (HR 2.320, 95% CI 0.903–5.957, P = 0.080) (Table 3).

**Table 3:** The adjusted risk of cerebrovascular mortality by local treatment groups

Variables	HR	95% CI	P
Entire cohort			
Surgery alone	1		
RT alone	1.703	1.398–2.075	< 0.001
Surgery plus RT	1.106	0.923–1.325	0.274
Aged 65–69 years			
Surgery alone	1		
RT alone	2.253	1.418–3.580	0.001
Surgery plus RT	1.352	0.862–2.121	0.188
Aged 70–74 years			
Surgery alone	1		
RT alone	1.591	1.161–2.179	0.004
Surgery plus RT	1.056	0.745–1.497	0.760
Aged ≥ 74 years			
Surgery alone	1		
RT alone	1.756	1.354–2.278	< 0.001
Surgery plus RT	1.179	0.938–1.481	0.158
Male			
Surgery alone	1		
RT alone	1.558	1.221–1.987	< 0.001
Surgery plus RT	1.098	0.871–1.384	0.429
Female			
Surgery alone	1		
RT alone	2.344	1.759–3.123	< 0.001
Surgery plus RT	1.104	0.844–1.445	0.471
Oral cavity, oropharynx, nasopharynx, and hypopharynx			
Surgery alone	1		
RT alone	2.519	1.881–3.374	< 0.001
Surgery plus RT	1.200	0.909–1.583	0.198
Other tumor sites			
Surgery alone	1		
RT alone	1.378	1.062–1.788	0.016
Surgery plus RT	1.058	0.833–1.344	0.646
SEER localized stage			
Surgery alone	1		
RT alone	1.682	1.430–1.979	< 0.001
Surgery plus RT	1.114	0.948–1.308	0.190
SEER regional stage			
Surgery alone	1		
RT alone	2.230	1.612–3.086	< 0.001
Surgery plus RT	1.146	0.852–1.543	0.368
SEER distant stage			
Surgery alone	1		
RT alone	4.862	2.005–11.792	< 0.001
Surgery plus RT	1.223	0.542–2.757	0.627
Married			
Surgery alone	1		
RT alone	1.502	1.201–1.878	< 0.001
Surgery plus RT	0.982	0.783–1.231	0.875
Unmarried			
Surgery alone	1		
RT alone	1.879	1.414–2.496	< 0.001
Surgery plus RT	1.165	0.901–1.506	0.243
No chemotherapy			
Surgery alone	1		
RT alone	1.682	1.410–2.008	< 0.001
Surgery plus RT	1.159	0.978–1.374	0.089
Chemotherapy			
Surgery plus RT	1		
Surgery alone	2.230	0.903–5.957	0.080
RT alone	1.779	1.210–2.615	0.003

## Discussion

In the current study, we analyzed the effect of RT on CVM in HNC survivors aged ≥ 65 years using a population-based cohort. Our results showed that older HNC survivors receiving RT alone had an increased risk of CVM compared to those receiving surgery alone or surgery plus RT. However, comparable CVM was found between surgery alone and surgery plus RT cohorts. Our findings are significant because CVM is one of the few long-term fatal toxicities of RT. Moreover, most previous studies utilized the CVD as study endpoint, no CVM.

An analysis using traditional RT technology included younger patients aged < 60 years from 1977 to 1998. It was found that any RT could significantly increase the risk of stroke (relative risk 10.1, 15-years risk of stroke 12.0%) [5]. A previous SEER-Medicare study included 6862 patients aged > 65 years (diagnosed between 1992 and 2002), with a median follow-up time was 3.2 years for those who survived, the 5-years incidence of cerebrovascular events was 14–19% [4]. A large cohort from Korea included 5570 HNC patients diagnosed between 2003 and 2005, 77.0% of patients receiving 2-dimensional RT, the 10-years ischemic cerebrovascular disease was approximately 5% [10]. A comprehensive study of 17 related literature published from 1981 to 2009 showed that the prevalence of transient ischemic attack and ischemic stroke after RT was 2.3–17.7% [13]. In our study, we included 16,923 HNC survivors with a median follow-up of 87 months, the 5-, 10-, and 15-years of CVM was 3.3%, 7.2%, and 11.5%, respectively. The study period of the present study is in the mode of contemporary radiation technology, although we use the CVM as the end-point of follow-up, it seems to be no significant difference in the incidence of cerebrovascular events or CVM among different RT technologies. In our study, 75% of patients had tumors developed in the oral cavity, pharynx, or larynx, which were prone to cervical lymph node metastasis and most of the radiation target volume included the neck.

Several studies have tried to answer the question regarding the relationship between RT and the risk of CVD or CVM in HNC. The results from the Korean cohort found that the receipt of RT increased the CVD risk by 40.8% than the surgery alone group [10]. However, RT did not increase the risk of CVM. In addition, they did not group the patients for definitive RT or postoperative RT. The study by Arthurs et al*.* included 14,609 patients from Canada, they found that RT alone (HR 1.70) or any RT cohort (HR 1.46) had a significantly higher risk of ischemic stroke compared to surgery alone [6]. However, they also did not group the patients for definitive RT or postoperative RT. In a study from Taiwan province of China included 10,172 HNC patients, they found that in patients aged < 55 years, RT alone had a higher risk of stroke event than those treated with surgery alone or surgery plus RT, and comparable risk of stroke event was found between surgery alone and surgery plus RT [11]. However, no statistical difference was found regarding stroke risk among different treatment modalities in those aged ≥ 55 years. A previous SEER-Medicare study included 6862 patients aged > 65 years (diagnosed between 1992 and 2002), the median follow-up months was 3.2 years for those who survived, they found that definitive RT but not adjuvant RT was associated with a higher CVD risk in these patients. The 10-years risk of cerebrovascular events was 34%, 25%, and 26% in those receiving RT alone, surgery plus RT, and surgery alone, respectively (4). However, laryngeal cancer patients were excluded from this study. In our study, approximately 25% of HNC patients were tumors located in the larynx, so the lack of analysis of the data regarding the larynx may have a potential impact on the results. Two studies from early-stage glottic cancer have found that the risk of CVD or CVM in patients receiving definitive RT was significantly higher than that in patients receiving surgery alone [14, 15].

In our study, older HNC patients receiving RT alone had increased CVM risk compared to those receiving surgery with or without RT. Our finding is consistent with the independent effect of RT on CVD or CVM in other cancers, including breast cancer [16, 17] and brain tumors [18, 19], which may expose high RT dose to carotid vessels or central arterial circulations.

Although patients receiving definitive RT have a higher risk of CVM (15-year 13.3%), we should note that the 15-year CVM of patients receiving surgery alone and surgery plus RT was 10.8% and 11.2%, respectively. In a previous large cohort study including 14,069 HNC patients at all ages (68.8% of patients were < 65 years old), the 15-year CVM of patients who underwent surgery alone was about 7.0% [6]. In addition, a previous SEER study showed a 10-year CVM of 0.64% in brain tumors patients who received postoperative RT [18], which was significantly lower than our study. Older HNC patients have more vascular risk factors compared to their younger counterparts, such as diabetes and hypertension affect the risk of CVM. Therefore, older age is a risk factor for the increased risk of CVM [7]. Moreover, it is important to note that complications caused by surgery, including fibrosis and adhesions, may also have adverse effects in the smaller vessels and carotid, leading to the increased risk of CVM in the surgery alone group [20]. Brown et al*.* found that the risk of carotid artery stenosis was 32% in patients who underwent neck lymph node dissection, which was significantly higher than those who had not (4%) [21]. Therefore, complete assessment of the modifiable risk factors and regular surveillance should be performed in older HNC patients who received different treatment strategies for early detection and intervention of CVD.

In terms of mechanism, RT-related atherosclerotic changes in the carotid arteries, hypertrophy of the intima, thickening and fibrosis of the endothelium and muscular wall thickening, and dysfunction of the elastic membrane may contribute to the vascular compromise and ischemic events in HNC patients [8, 22, 23]. However, patients who were treated with surgery plus RT did not associate with a higher CVM risk in our study. The reduction in the total postoperative RT dose to the head and neck regions may explain this phenomenon [4, 24, 25]. Haynes et al*.* included 413 patients who received RT to the head and neck. A total of 20 patients developed experienced CVD and all of these 20 patients had received more than 60 Gy, which suggested a significant link between RT dose and the development of CVD [26]. The study from Duke Cancer Institute also found that the higher risk of carotid artery stenosis was associated with a higher RT dose to carotid bulb plus 2 cm [27]. In addition, the dose to the carotid arteries was also had an independent effect on the risk of CVD (HR = 1.11, P < 0.001) [28]. In the current clinical practice, HNC patients who received postoperative RT typically received total RT doses of approximately 54–63 Gy (60–66 Gy in patients with adverse features), whereas patients treated with definitive RT typically received 66–70 Gy [29]. The previous study including older breast cancer patients reported no increased risk of CVD in patients receiving postoperative supraclavicular irradiation with an RT dose typically between 45 and 50 Gy [30]. The RT dose is highest in those receiving definitive RT or radiochemotherapy. Therefore, we can make a hypothesis that the dose threshold for developing clinically significant CVD may be between 66 and 70 Gy and the incidence of CVD or CVM is a dose-dependency. For HNC patients, the effect of surgery or postoperative RT on vascular stenosis is considered insignificant, while definitive RT contributed to a higher risk of CVD or CVM.

The mean time for CVM was shorter in the RT alone group than those in the surgery alone group or surgery plus RT group (52 months vs. 56–60 months). The study by Lee et al*.* showed a mean time to CVD was 64 and 62 for those treated with non-RT and RT groups, respectively [10]. Since the chronic damage to the artery caused by RT may be long-term, the incidence of CVD or CVM after RT is still possible during the long-term follow-up. Therefore, it is necessary to perform a long-term follow-up and regular screening of CVD risks for HNC patients who received definitive RT. However, the CVD risk caused by RT should not be potentially exaggerated or overestimated beyond the hazards by traditional risk factors.

Several limitations warrant mention in this study. First, SEER only records CVM but not CVD as isolated events. Second, the details of RT, including RT technology, RT dose, fractionation utilized, specific RT fields, and target volume definition are not captured in the SEER database. Therefore, treatment groups in the present study may not reflect completely homogeneous therapeutic strategies. Third, it is a large difference in RT fields and dosages if an early-stage laryngeal cancer has been treated by RT alone (for example 66 Gy) or a locally advanced laryngeal cancer has been treated by radiochemotherapy (70 Gy or above). Therefore, the effects of different treatment strategies among HNC patients on the CVM may be different by stage. However, we also found a significant effect of definitive RT on the increase of CVM regardless of the SEER stage in the stratified analyses. Interestingly, we found that with the increase in the SEER stages, the effect of definitive RT on CVM increased significantly. Moreover, a previous SEER-Medicare study including 6862 HNC patients (age > 65 years), and approximately 40% of patients had comorbidities, patients with severe comorbid disease were more likely to receive RT alone [4]. Due to the lack of comorbidity status in the SEER database, it is worth noting that unidentified confounding factors may exist for the relation to CVM in patients receiving RT alone. Future studies with more detailed RT and comorbidity status are required to further rule out potential confounding factors to determine the true magnitude of relation in patients receiving RT alone. Finally, information regarding the comorbidities, alcohol consumption, and smoking status are also not available in the SEER database. However, a previous compliance study on the HNC RT trials found that nearly 90% of patients completely received the prescribed RT doses [31].

## Conclusion

In conclusion, our study indicates that definitive RT can increase the risk of CVM among older HNC survivors. Long-term follow-up and regular screening for CVD are required for HNC patients who received definitive RT to decrease the risk of CVM. It is necessary to further optimize the RT technology and the delineation of the target volume to minimize the dose to the carotid artery to reduce the risk of CVM.

## Data Availability

Any request for data and material may be sent to the corresponding author.

## Abbreviations

HNC: Head and neck cancer
RT: Radiotherapy
CVD: Cerebrovascular disease
CVM: Cerebrovascular mortality
SEER: Surveillance, Epidemiology, and End Results
US: United States
HR: Hazard ratio
CI: Confidence interval
